# Contrast-enhanced ultrasonography of the pancreas shows impaired perfusion in pancreas insufficient cystic fibrosis patients

**DOI:** 10.1186/s12880-018-0259-3

**Published:** 2018-05-15

**Authors:** Trond Engjom, Kim Nylund, Friedemann Erchinger, Marcus Stangeland, Birger Norderud Lærum, Martin Mézl, Radovan Jiřík, Odd Helge Gilja, Georg Dimcevski

**Affiliations:** 1 Department of Clinical Medicine, University of Bergen, Bergen, Norway; 2 Department of Medicine, National Centre for Ultrasound in Gastroenterology, Haukeland University Hospital, 5021 Bergen, Norway; 3 Department of Medicine, Voss Hospital, Voss, Norway; 4 Department of Clinical Science, University of Bergen, Bergen, Norway; 5 LHL-clinics Bergen, Bergen, Norway; 6 Department of Biomedical Engineering, Faculty of Electrical Engineering and Communication, Brno University of Technology, Brno, Czech Republic; 7 Institute of Scientific Instruments of the Czech Academy of Sciences, Brno, Czech Republic

**Keywords:** Contrast enhanced ultrasound, Pancreas, Perfusion, Exocrine pancreatic function, Cystic fibrosis

## Abstract

**Background:**

Perfusion assessment of the pancreas is challenging and poorly evaluated. Pancreatic affection is a prevalent feature of cystic fibrosis (CF). Little is known about pancreatic perfusion in CF. We aimed to assess pancreatic perfusion by contrast-enhanced ultrasound (CEUS) analysed in the bolus-and-burst model and software.

**Methods:**

We performed contrast enhanced ultrasound of the pancreas in 25 CF patients and 20 healthy controls. Perfusion data was analysed using a dedicated perfusion model providing the mean capillary transit-time (MTT), blood flow (BF) and blood-volume (BV). CF patients were divided according to exocrine function.

**Results:**

The pancreas insufficient CF patients had longer MTT (*p* ≤ 0.002), lower BF (*p* < 0.001) and lower BV (*p* < 0.05) compared to the healthy controls and sufficient CF patients. Interrater analysis showed substantial agreement for the analysis of mean transit time.

**Conclusion:**

The bolus-and-burst method used on pancreatic CEUS-examinations demonstrates reduced perfusion in CF patients with pancreas affection. The perfusion model and software requires further optimization and standardization to be clinical applicable for the assessment of pancreatic perfusion.

**Electronic supplementary material:**

The online version of this article (10.1186/s12880-018-0259-3) contains supplementary material, which is available to authorized users.

## Background

Non-invasive measurements of pancreatic perfusion have been performed by contrast-enhanced ultrasound (CEUS) [1–5], perfusion Computer tomography (CT) [6, 7] and magnetic resonance imaging (MRI) [8]. Attempts at an in-vivo reference standard have been made using hydrogen gas clearance method under laparoscopy [9] and endoscopy [10]. In a recent review of the methods, not including CEUS, Tsushima et al. reported median value of normal pancreatic perfusion from several studies around 100 mL/min/100 mL of pancreatic tissue, with individual values ranging from 38.4 to 356 mL/min/100 mL [7]. A standard for the clinical use of CEUS dealing with pancreatic lesions has been suggested [4]. However, a clinical application of pancreatic perfusion measures has not been established.

CEUS by gas-filled microbubbles is commonly used to assess relative perfusion parameters in various organs [4, 11–13]. In the gut CEUS modelling of perfusion can be used for separating inflammatory from fibrotic processes [12, 14]. In the pancreas perfusion can be useful in a clinical setting such as characterizing tumours [2, 15] and in the evaluation of other focal pancreatic lesions [16]. One study was able to demonstrate reduced perfusion in chronic pancreatitis patients compared to healthy controls [10], and attempts have been made to define perfusion changes in early chronic pancreatitis [5].

Most integrated tools on the ultrasound scanners provide models to calculate parameters from the time intensity curve [2, 10, 14, 15]. Often the models are inaccurate, and analysis is performed offline on exported data sets [14, 17]. A unique feature of microbubbles is that they can be cleared from the bloodstream using a burst of ultrasound with a high mechanical index. Recently, Jirik et al. developed a method for estimating absolute perfusion parameters; the bolus-and-burst technique [18]. The pharmacokinetic model used allows the estimation of the mean capillary transit time (MTT), the blood volume (BV), and also blood flow (BF) using the central volume theorem, BF = BV/MTT [18, 19]. The integral of the time-intensity curve in a vessel (artery or vein) is used for scaling the data and calculate absolute values for blood flow and blood volume. The model has earlier been related to pathological findings and clinical outcome data in the intestines of patients with Crohn’s disease [12]. We recently evaluated the inter observer and inter system quality of the model in the pancreas [20].

Patients with cystic fibrosis (CF) develop pancreatic damage as a result of defective ductal and acinar pancreatic secretion [21, 22]. The main pathological findings in the affected CF pancreas are homogenous atrophy, fibrosis and fatty infiltration, whereas the more focal features of chronic pancreatitis are not frequently seen [21, 23]. The microvascular changes and perfusion characteristics in CF are not well described in literature. Autopsy studies of chronic pancreatitis patients have shown marked reduction in pancreatic vasculature with reduced number, volume and calibre changes of the vessels [24]. It is assumed that the same reduction of microvascular density is present in the affected CF pancreas. The possibility of precise pancreas phenotype characterization, and a marked, homogenous pathological difference between normal and affected tissue, makes CF patients a good model population for examining pancreas perfusion.

The aim of this study was to assess absolute perfusion parameters in the pancreas using bolus-and-burst technique [12, 18, 19] in patients with CF and healthy controls. Furthermore, the interrater agreement of the model-analysis was evaluated.

## Methods

### Subjects

During a 4-year period (December 2010–May 2014), CF patients aged > 15 years attending regular follow up in the CF clinic were offered a detailed evaluation of the pancreas in this prospective observational study. The CF diagnosis was defined according to the present diagnostic criteria for CF in the cystic fibrosis foundation consensus report [25, 26]. A group of healthy controls was also included. Inclusion criteria for the control group were absence of abdominal symptoms and disease. Subjects with insufficient sonographic visualisation of the pancreas, extensive respiratory movements or technical flaws in the recorded perfusion/reperfusion phases were excluded retrospectively.

### Patient characteristics

Patient records were reviewed and all subjects were interviewed. Age and sex of the patient, body mass index, *CFTR* mutation status and sweat-test values (Cl^−^) were documented.

### Transabdominal ultrasound

The subjects were fasting > 4 h. A GE Logic E9 scanner with a 1-5 MHz curvilinear-probe was used (General Electric medical systems and primary care diagnostics, Milwaukee, WI, USA). Scanning of the pancreas was performed with the subjects in supine position, using a transverse or oblique epigastric probe position. An intravenous bolus of 1.5 mL SonoVue® contrast agent (Bracco, Milan, Italy) was given over 2 s followed by a bolus of 10 ml saline over 4 s. The dual view containing both the B-mode and the contrast image was used for the acquisition. Recordings were acquired for 90 s with the following settings: Dynamic range 66, 9 frames per second, probe frequency 4 MHz and mechanical index: 0.10. The focus position was placed at the deepest point of the pancreas. When the contrast intensity reached a steady state (after 45 s) a high-power pulse sequence was applied to burst the bubbles, and the replenishment phase was recorded for 45 s. Recordings were stored as Digital Imaging and Communications in Medicine (DICOM)-files for later analysis.

### Image processing and analysis

#### Exclusion of datasets

To ensure relevant and good quality data, a strict exclusion procedure was applied. Datasets with generally insufficient imaging quality or particularly with disturbances in the reperfusion phase were excluded prior to the final analysis. Exclusions were made blinded to patient characteristics. Post analysis exclusions of outliers represented by MTT > 15sek were performed to exclude non-physiological values from the analysis.

### Image processing and analysis

**Fig. 1:**
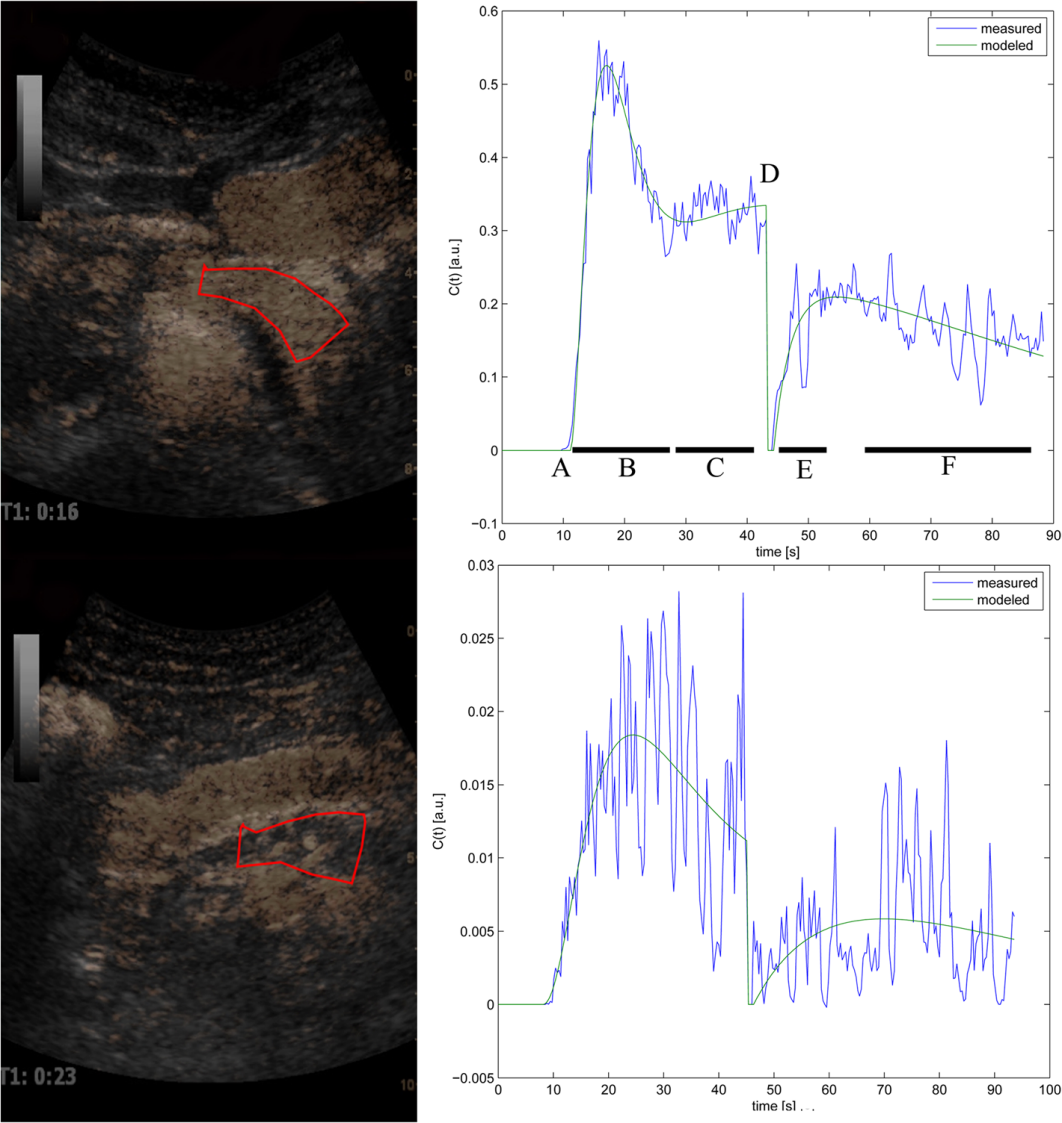
Analysis and perfusion curves. The figure displays contrast images from with a chosen region of interest (Red encirclement) in the body/tail of the pancreas in a sufficient (upper) and insufficient (lower) CF patient. The ROIs were placed by guidance from the B-mode image. Mark that the ROI in the insufficient patient is placed over an atrophic and poorly perfused pancreas. The highly perfused structure anterior to the pancreas is the ventricular wall. The perfusion curves with different phases are presented to the right the measured (blue) and modelled (green) curves. A: Arrival of contrast. B: First pass of contrast bolus. C/F: Linear decay phases. D: Burst. E: replenishment phase. Curves are presented before scaling to the arterial input, thus values are in arbitrary units and not directly comparable. (C(t): Concentration over time. [a.u]: Arbitrary units

Contrast images with chosen ROIs and corresponding perfusion curves are illustrated in Fig. 1.

### Manual motion correction

To reduce the size of the data sets and thus computational load, the sequences were automatically subsampled to 3 frames per second. Furthermore, a manual single frame exclusion procedure and movement correction was performed using a perfusion analysis calculation-software (DCE-US, http://info.isibrno.cz/perfusion/) implemented in MATLAB (Version R2014a, Mathworks Inc., Massachusetts, USA) as previously described [27]. The calculations in the DCE-US software were performed within a defined region of interest (ROI) in the pancreas after the motion correction and frame exclusion. The ROI was chosen in a region of the pancreas with minimal residual motion and other artefacts. The size and form of the ROI had to be adjusted according to this, excluding the possibility of a standardized ROI for all patients. An anatomical location in the head or body of the pancreas was preferred, but sample quality had priority over exact standardization of anatomical location. For the scaling procedure an artery close to the chosen ROI (mainly superior mesenteric or gastroduodenal artery) was identified. If necessary a second motion correction process was performed before a ROI was drawn and the integral of the arterial tissue concentration curve calculated.

The manual motion correction and perfusion analysis was performed by clinicians experienced (> 5 years) with pancreatic ultrasound and blinded to knowledge of pancreatic function and clinical data.

### Perfusion analysis

In Fig. 1 we present the perfusion curves for selected parts of the pancreas in two patients. The complex mathematical calculations in the bolus-and-burst perfusion-analysis model are described in detail by Jirik et al. [18, 19]. It provides estimates of the physiological parameters blood volume (BV [mL/100 mL]), mean capillary transit time (MTT [s]) and blood flow (BF [mL/min/100 mL tissue]) calculated as BF = BV/MTT*60. The perfusion model includes a scaling factor required for absolute quantification of blood volume and flow [18, 19] derived from the area under the curve of the arterial tissue concentration curve.

### Interrater analysis

Before analysis the DICOM files were randomized, using a web based free-ware, Research Randomizer [28]. The perfusion analysis was performed by two observers (KN and TE). The results from observer 2 were used for interrater analysis only.

### Exocrine pancreas function

We assessed exocrine pancreatic function by a secretin stimulated, short endoscopic function test described elsewhere [29, 30]. Faecal elastase-1 was measured by a commercial analysis kit (ScheBo, Biotech, Giessen, Germany). The CF patients were defined as pancreas sufficient by faecal elastase > 100 μg/g or duodenal bicarbonate > 80 mmol/L.

### Statistical analysis

Statistics were calculated in SPSS statistics 22 (IBM SPSS Statistics, New York, USA) and SigmaPlot 11, (Systat Software Inc., San Jose, CA, USA). Normal distribution of the samples was tested by Kolmogorov-Smirnov test. The results are presented as median values with IQ range. Simple comparisons between groups were made Mann-Witney U-test. Accuracy data are calculated from receiver operator curves (ROC). Variance is expressed through 95% confidence intervals. 5% level of statistical significance was used. Correlation was calculated using Pearson’s correlation coefficient. Interrater reliability was calculated as intra-class correlation coefficients (ICC) in a random, two-way analysis. The ICC has values between 0 and 1 and is considered poor if 0–0.2, fair if 0.2–0.4, good if 0.4–0.75 and excellent if > 0.75. The scaled data were analysed according to consistency. Agreement was defined according to Landis and Koch [31]: 0  =  no agreement, 0 - 0.20  =  slight agreement, 0.21–0.40  =  fair agreement, 0.41- 0.60  =  moderate agreement, 0.61–0.80  =  substantial agreement and 0.81–1  =  almost perfect agreement. Bland-Altman plots were drawn. Power and number of patients at baseline are calculated based on the following assumptions: The smallest difference between the groups rejecting the null hypothesis is estimated to 35%. The worst case standard deviation is chosen 25%. Sample sizes of 14 patients in each group are expected to give the desired power of at least 0.80.

## Results

### Inclusion

**Fig. 2:**
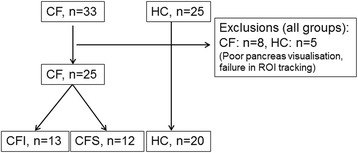
Inclusion flow chart. The figure displays the inclusion flow chart and exclusions. CF: Cystic fibrosis. CFI/CFS: Cystic fibrosis insufficient/ sufficient. HC: Healthy controls

The inclusion flow chart is displayed in Fig. 2. We examined 33 CF patients and 25 healthy controls according to the protocol. We excluded 8 CF patients and 5 controls due to poor pancreatic ultrasound visualisation, or failure to track the same region of interest throughout the examination. Accordingly, we present results from 25 CF patients and 20 healthy controls (HC). When sorted by exocrine pancreatic function, patient groups were divided as follows: Cystic fibrosis; pancreatic insufficient (CFI, *n* = 13) and cystic fibrosis; pancreatic sufficient (CFS, *n* = 12). Observer 2 failed to achieve analysis for four of 45 subjects analysed by observer 1. Further three analyses were excluded from the analysis performed by observer 2 due to non-physiological values (MTT > 15 s), leaving 38 analyses for the interrater agreement analysis. Demographic data and data for exocrine function are displayed in Table 1. The control group was slightly older and contained more females than the CF groups (*p* < 0.05).

**Table 1:** Demographic data and exocrine function

	CFI (*n* = 13)	CFS (*n* = 12)	HC (*n* = 20)	*p*
Age	21 (16–52) ^*^	21 (16–70)^*^	26 (18–66) ^*^	
Gender (♀/♂)	6 / 7	6 / 6	13 / 7	
Body mass index	21 (19–23)	22 (21–25)	22 (20–25)	
Sweat [Cl^−^]	113 (100–130)	72 (66–78)	–	***
F-Elastase (μg/g)	0 (0–2)	571 (512–612)	–	***
D-bicarbonate (meq/L)	11 (11–19)	118 (96–130)	–	***

Values are expressed as medians (IQ range) unless otherwise stated (*Median (Range) (CFI/ CFS: Cystic fibrosis insufficient/ sufficient, HC: Healthy controls). *: < 0.05, **: < 0.01, *** < 0.001)

### Perfusion parameters and exocrine function

We calculated BF in mL/min/100 mL, BV in mL/100 mL and MTT in seconds in all three patient groups based on values from of observer 1.

**Fig. 3:**
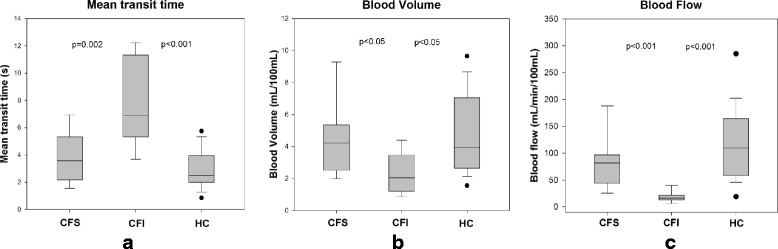
Perfusion parameters. Box plots for mean capillary transit time (MTT, panel **a**), blood volume (BV, panel **b**) and blood flow (BF, panel **c**) in patients and healthy controls

The results are displayed in Table 2 and Fig. 3. Pancreatic insufficient CF patients had significantly longer MTT (*p* ≤ 0.002), lower BF (*p* < 0.001) and lower BV (p < 0.05) compared to healthy controls and pancreatic sufficient CF patients. Pearson correlations between duodenal bicarbonate and perfusion parameters gave the following results: MTT: *r* = − 0.58, *p* = 0.008, BV *r* = 0.45, *p* = 0.046, BF: *r* = 0.44, *p* = 0.05. Best differentiation between CFI and CFS was made using MTT and blood flow. A difference between pancreas sufficient CF patients and healthy controls was not observed. Calculated blood flow values in our healthy control group were comparable to values obtained by other methods [7, 9].

**Table 2:** Perfusion parameters

	CFI (*n* = 13)	CFS (*n* = 12)	HC (*n* = 21)	*p*
MTT (s)	6.9 (5.3–11.3)	3.6 (2.2–5.3)	2.5 (2.0–4.0)	**
BV (ml/100 mL)	2.1 (1.2–3.5)	4.2 (2.5–5.3)	3.9 (2.7–7.1)	*
BF (ml/min/100 ml)	17 (13–22)	82 (44–97)	110 (59–165)	***

Values are expressed as median (IQ range). There is no difference between CFS and HC group. Concerning difference in BF for these two groups, the power is below desired level

### Diagnostic accuracy

**Fig. 4:**
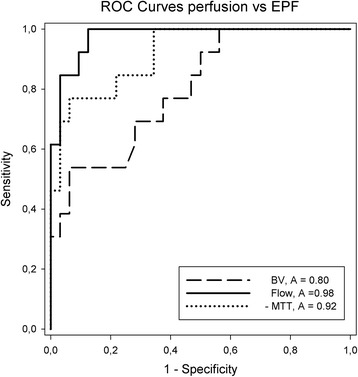
Diagnostic accuracy. Receiver operator curve for the perfusion parameter vs exocrine pancreatic failure. ROC: Receiver operator curve, EPF: Exocrine pancreatic function, A: Area under curve, MTT; Mean capillary transit time. BV: Blood volume

We also calculated receiver operator curves (Fig. 4) expressing the diagnostic quality of the three parameters in predicting exocrine pancreatic failure. Sensitivity and specificity for the suggested cut-offs are displayed in Table 3.

**Table 3:** Diagnostic accuracy

	Sensitivity	Specificity	Cutoff	Accuracy
MTT	0.77 [0.46–0.95]	0.78 [0.60–0.91]	5.0	0.92
BV	0.69 [0.39–0.91]	0.72 [0.53–0.86]	3.0	0.80
BF	1.0 [0.75–1.0]	0.88 [0.71–0.96]	45	0.98

Table displaying values for diagnostic accuracies for the corresponding cut-offs. Values in means [95%CI]

### Interrater quality

**Fig. 5:**
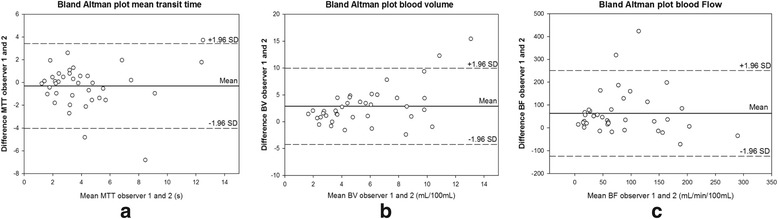
Interrater agreement. Bland Altman plots displaying the differences between the values for observer 1 and observer 2 for mean transit time (panel **a**), blood volume (panel **b**) and blood flow (panel **c**) in patients and healthy controls. (MTT; Mean capillary transit time. BV: Blood volume, BF: Blood flow, SD: Standard deviation, mL: Millilitres, s: seconds)

The parallel results from observer 1 and 2 were compared. The agreement for all values is presented as Bland-Altman plots in Fig. 5. Inter-correlation coefficient (ICC [95%CI]) was calculated for all three parameters and demonstrated excellent agreement for MTT (ICC 0.78 [0.62, 0.88]) where the differences between the observations were non-different from zero and linear regression detected no bias through the range of MTT. For the two other parameters the agreement was moderate (BV: ICC 0.44 [0.14, 0.67], BF: ICC 0.48 [0.19, 0.69]. A single variable t-test demonstrated that the difference between the observed values for these parameters differs from zero (*p* < 0.05), and linear regression detected a fixed bias with a higher values in the calculations from observer 2.

## Discussion

We calculated absolute pancreatic perfusion parameters using the bolus-and-burst method applied on data from contrast-enhanced ultrasound of the pancreas in CF patients and healthy controls. The results were related to exocrine pancreatic function. We demonstrate two main findings: First, we show that pancreatic perfusion in pancreatic insufficient CF patients is reduced compared to pancreatic sufficient patients and healthy controls. Secondly we found that the reduced perfusion calculated in our absolute perfusion model in CF patients predicts exocrine pancreatic insufficiency in CF with an acceptable diagnostic accuracy. Accordingly, CEUS with absolute perfusion analysis in the bolus-and burst model may non-invasively differentiate between healthy pancreatic tissue and exocrine insufficient pancreatic tissue due to CF. For the inter-rater quality of the analysis process we find excellent agreement for MTT, whereas the two other parameters demonstrate a fixed bias between the raters.

Presently, this is the only study describing perfusion aspects in the CF pancreas. The CF pancreas was chosen to evaluate the CEUS bolus-and-burst method due to the clear and homogenous discrimination between healthy and abnormal tissue. The exocrine function in our population was either normal or very low. This finding was distinctly reflected in the distribution of the bicarbonate levels between individuals with a healthy or affected pancreas. By the combination of faecal elastase and endoscopic short test to define exocrine pancreatic function we provide a strict and accurate definition of pancreas sufficiency [29]. Few results in the intermediate range reduce the value of correlation studies between perfusion and exocrine function parameters. However, the perfusion parameters still demonstrate acceptable correlation to exocrine pancreatic function.

The literature describing the vascularity of the affected CF pancreas is lacking, but due to the described severe fatty infiltration and progression towards atrophy, fibrosis and degeneration of normal anatomy [23], we assume that the vascular density in the pancreas is reduced. This hypothesis fits our findings where measured blood volume and the calculated blood flow are reduced and the transit time is increased in the affected pancreas. The clinical application of this finding in CF diagnostics is possibly limited, but the ability to differ between tissues with normal and reduced perfusion may be more relevant in other diseases. The clinical application of CEUS in the differential diagnostics of pancreatic tumours is already established [4]. Furthermore, particularly in chronic pancreatitis the gland may be focally destroyed due to obstructive causes. In this setting, the discrimination between vital tissue connected to recent changes and more irreversibly destroyed tissue due to longstanding changes could be relevant for the therapeutic decision process.

Contrast-enhanced ultrasound of the pancreas infers several challenges both in image quality, reproducibility and standardisation. In this study we exported the image data and used a non-integrated perfusion analysis tool instead of the widespread integrated tools. The advantages of tools integrated in the scanners are better availability and clinical feasibility. However, questions have been raised about the variability of these tools in different scanners and the validity of the mathematical model using log-converted time-intensity data [18, 19, 32]. One study achieved acceptable improvements in interrater reproducibility for a range of perfusion parameters both in vivo and vitro using the bolus method and taking into account the arterial input function and a mathematical model based on deconvolution [32].

In the model from Jirik et al., absolute perfusion can be calculated using a combination of the bolus and the burst-replenishment methods. The scaling of the data to the area under the curve of the TIC from a local artery enables accurate estimates of both time and amplitude related variables such as mean transit time and blood volume, respectively [18, 19]. The disadvantage of the chosen method compared to the integrated models, is the complexity of the analysis which includes several steps of motion compensation, removal of off-plane images, ROI selection and selection of small, difficult-to trace arteries for scaling. The process is manual and time-consuming, and contains several steps prone to introduce variability. Both raters demonstrated reduced perfusion and prolonged transit time in the affected CF pancreas, but our analysis revealed less than desired quality in interrater agreement. The methodological complexity probably explains the variation between the observers. Although the model infers complicated mathematics and the software still is in beta-version, we advocate that the advantages of absolute perfusion parameters and transferability between different ultrasound systems justify further validation of this model.

### Limitations of study

We have demonstrated previously that pancreas sonography is feasible in most CF patients [33]. The localisation of the pancreas deeply in the abdomen behind other air-containing organs like the colon, the ventricle and the duodenum can cause permanent or intermittent disturbances. This affects an analysis dependent on observation over time. To deal with these challenges we excluded patients with poor image quality, and performed motion correction and frame exclusions as described above. This introduces possible exclusion bias. The number of subjects in each group after exclusions was slightly below calculations for desired power, thus non-significant differences should be interpreted with caution.

Blinding the operators to information on subject characterisation and exclusions in the pre-analytic stage were measures taken to reduce the risk of such biases. We acknowledge that a high number of exclusion is a limitation of the clinical usefulness of the method. However, we argue that these measures make the final perfusion assessment less prone to imaging disturbances and improve the physiological reliability of the results.

In a clinical setting, the usefulness of the method depends on adequate standardisation and quality of the CEUS recordings, and subsequently high quality motion correction. Difficulties in standardization of position, form and size of the ROIs may be a limitation introducing variations.

Forthcoming software improvements to reduce the number of manual steps and to automatize tissue recognition will most likely increase interrater agreement.

## Conclusions

We found that CEUS in the chosen absolute perfusion model may non-invasively differentiate between healthy and affected pancreatic tissue due to CF. To our knowledge there are no other studies estimating perfusion in the CF pancreas or relating the perfusion to exocrine function. A good clinical standard for the evaluation of pancreatic tissue perfusion is presently non-existing. The CEUS acquisition and perfusion estimation in the bolus–and-burst model requires further optimization to be widely clinical applicable for the assessment of pancreatic perfusion.

## Additional file

Additional file 1: Anonymized data file displaying the background data used in the article. (XLSX 16 kb)

## Abbreviations

BF: Blood flow
BV: Blood-volume
CEUS: Contrast enhanced ultrasound
CF: Cystic fibrosis
CFI/CFS: Cystic fibrosis pancreas insufficient/sufficient
CFTR: Cystic fibrosis transmembrane receptor protein
CT: Computer tomography
DICOM: Digital Imaging and Communications in Medicine
HC: Healthy controls
ICC: Intra-class correlation coefficients
IQ-range: Interquartile range
MRI: Magnetic resonance imaging (MRI)
MTT: Mean capillary transit-time
ROI: Region of interest
SD: Standard deviation
TIC: Time intensity curve
